# Stratifying risk of recurrence in stage II colorectal cancer using deregulated stromal and epithelial microRNAs

**DOI:** 10.18632/oncotarget.3225

**Published:** 2015-03-07

**Authors:** Marc D. Bullock, Karen Pickard, Richard Mitter, A. Emre Sayan, John N. Primrose, Cristina Ivan, George A. Calin, Gareth J. Thomas, Graham K. Packham, Alex H. Mirnezami

**Affiliations:** ^1^ University of Southampton Cancer Sciences Division, Somers Cancer Research Building, Southampton University Hospital NHS Trust, Tremona road, Southampton, UK; ^2^ Bioinformatics Unit, London Research Institute, Cancer Research UK, London, UK; ^3^ Center for RNA Interference and Non-Coding RNAs, The University of Texas MD Anderson Cancer Center, Houston, TX, USA; ^4^ Department of Surgery, Southampton University Hospital NHS Trust, Southampton, UK

**Keywords:** Colorectal cancer, microRNA, stroma, tumor microenvironment

## Abstract

MicroRNAs (miRNAs) enable colonic epithelial cells to acquire malignant characteristics and metastatic capabilities. Recently, cancer relevant miRNAs deregulated during disease progression have also been identified in tumor-associated stroma.

By combining laser-microdissection (LMD) with high-throughput screening and high-sensitivity quantitation techniques, miRNA expression in colorectal cancer (CRC) specimens and paired normal colonic tissue was independently characterized in stromal and epithelial tissue compartments. Notably, deregulation of the key oncogene miR-21 was identified exclusively as a stromal phenomenon and miR-106a, an epithelial phenomenon in the malignant state.

MiRNAs identified in this study successfully distinguished CRC from normal tissue and metastatic from non-metastatic tumor specimens. Furthermore, in a separate cohort of 50 consecutive patients with CRC, stromal miR-21 and miR-556 and epithelial miR-106a expression predicted short disease free survival (DFS) and overall survival (OS) in stage II disease: miR-21 (DFS: HR = 2.68, *p* = 0.015; OS: HR = 2.47, *p* = 0.029); miR-556 (DFS: HR = 2.60, *p* = 0.018); miR-106a (DFS: HR = 2.91, *p* = 0.008; OS: HR = 2.25, *p* = 0.049); combined (All High vs. All Low. DFS: HR = 5.83, *p* = 0.002; OS: HR = 4.13, *p* = 0.007).

These data support the notion that stromal as well as epithelial miRNAs play important roles during disease progression, and that mapping patterns of deregulated gene expression to the appropriate tumor strata may be a valuable aid to therapeutic decision making in CRC.

## INTRODUCTION

The majority of deaths from CRC are not caused by primary tumors, which are often resectable, but by metastatic disease, to which the most troublesome and intractable symptoms can be attributed and to which most patients eventually succumb [1, 2]. Despite increasing use of metastectomy and chemotherapy over time, the proportion of patients with metastatic disease who survive beyond 2 years remains as low as 28% [3]. Without treatment median survival is 8 months [4], but the addition of chemotherapy and newer targeted therapies may enhance this figure to 12 and 16 months respectively [4, 5].

The emergence of targeted therapies has been made possible through a greater understanding of the molecular characteristics of CRC. Although the results of clinical trials have been disappointing, extending survival by only a few months [6, 7], these studies offer proof-of-principle that therapeutic strategies translated from a better understanding of the mechanisms of disease can be applied to CRC. Equally significant is the prospect that molecular profiling may lead to more personalized patient care as therapeutic approaches become increasingly tailored to individual patient's needs [8].

Consequently, in recent times, the identification of biomarkers capable of predicting outcome or response to therapy and the identification of putative therapeutic molecular targets in CRC have become important priorities [9, 10].

MicroRNAs (miRNAs) are a class of highly conserved non-coding RNA molecules with essential regulatory and homeostatic cellular functions. They are deregulated in all cancers examined to date, and aberrant expression in CRC promotes both malignant transformation and metastatic progression [11–13]. Crucially, tumors of different developmental origin and tumors of progressive pathological stage exhibit unique patterns of deregulated miRNA expression, which has prompted extensive research to explore the potential clinical roles of miRNA profiling from whole tumor sections, lymph nodes, metastases, serum and even feces of patients with CRC [11, 14].

Emerging data suggests that metastasis relevant miRNAs are not simply confined to malignant epithelial cells but are also present in the stromal tissue surrounding a tumor. Deregulated stromal miRNAs may play an important role during metastatic progression by regulating the phenotype of cancer associated stromal cells [15–17] and through paracrine effects resulting from exosomal intracellular transfer [18, 19]. They may also provide valuable prognostic and diagnostic information during CRC progression as well as opportunities for novel pharmacological intervention [20, 21]. However, systematic studies characterizing discrete patterns of deregulated miRNA expression in CRC stroma and epithelium are lacking.

The purpose of the current study is to identify miRNAs deregulated in the CRC stroma and epithelium at different clinically relevant stages and to examine their clinical utility as biomarkers of disease progression.

## RESULTS

### Stromal and epithelial tissue compartments produce distinct patterns of miRNA deregulation in CRC

Most cancer research is focused on tumor cells; however recent developments suggest that molecular events in surrounding stromal cells may also play key roles during disease progression. To identify stromal miRNAs deregulated in CRC we conducted QuantimiR™ qPCR microarray analysis using RNA collected from 10 patients (5 Duke's A and 5 Duke's C). LMD stroma from primary CRC tissue specimens frozen in liquid nitrogen at the time of surgery was compared with stroma from paired, uninvolved, proximal colonic tissue from the same patient.

**Figure 1:**
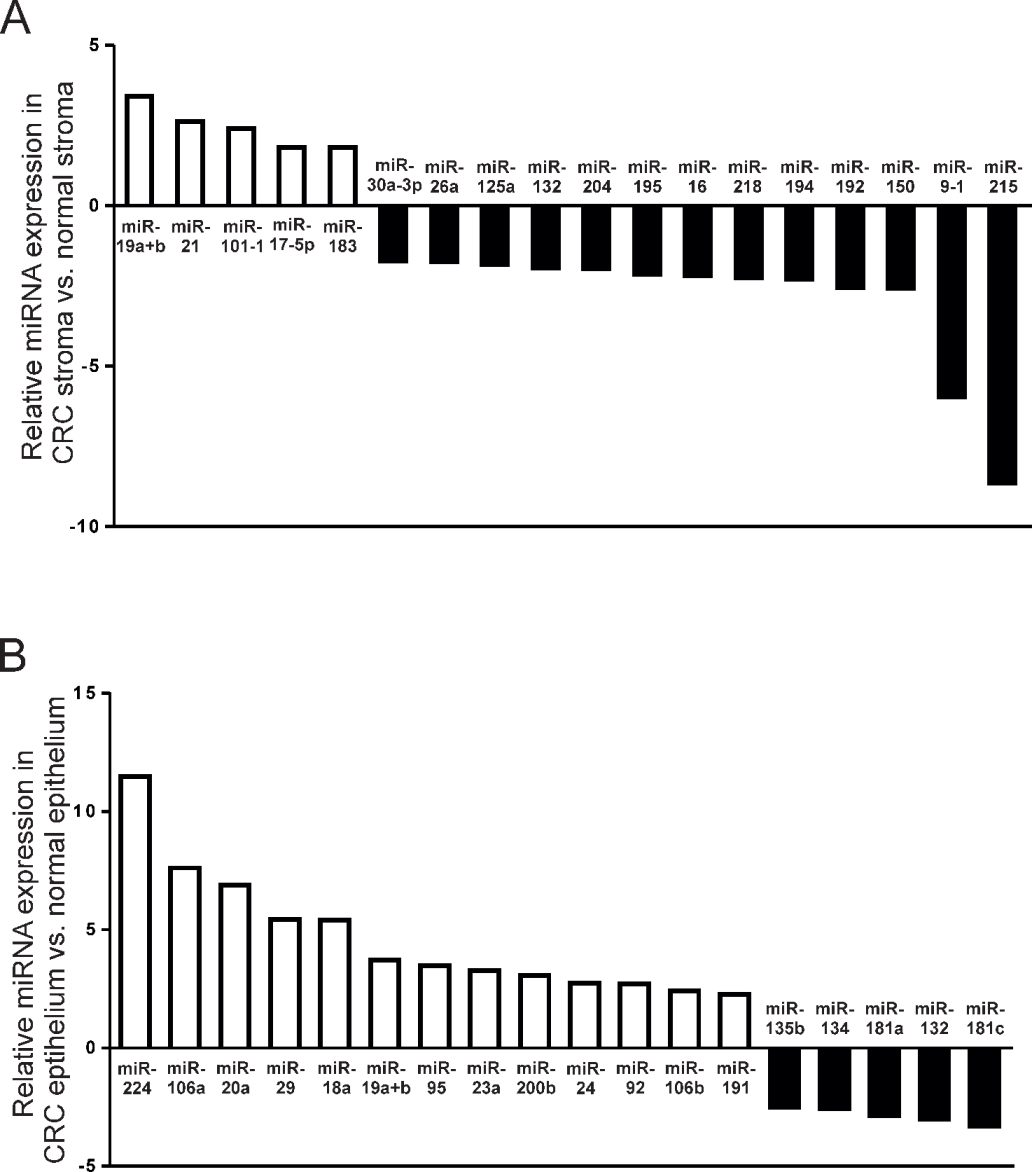
MiRNAs significantly differentially expressed by a factor > 2 (*p* < 0.05) in (A) CRC stroma vs. paired normal colonic stroma; and (B) CRC epithelium vs. paired normal colonic epithelium QuantimiR™-qPCR analysis was performed using total RNA extracted from fresh-frozen LMD tissue from 10 patients with CRC.

In total, 5 miRNAs were significantly upregulated and 13 miRNAs significantly downregulated in CRC stroma compared with paired normal tissue (Figure 1A). Amongst the miRNA candidates upregulated more than X2-fold were miR-19a and -19b and miR-21, which are recognized oncogenes [22–26].

Subsequent profiling of LMD CRC epithelium from the same patient cohort revealed an entirely distinct pattern of miRNA deregulation compared with stromal tissue. In contrast to stroma, 13 epithelial miRNAs were significantly upregulated and 5 miRNAs significantly downregulated in CRC epithelium compared with paired normal colonic epithelium (Figure 1B). Except for miR-19a and 19b, upregulated more than X2-fold in both CRC epithelium and associated stromal tissue, no other miRNA candidates were deregulated in both tumor strata.

These data emphasize the clear biological distinctions between stromal and epithelial tissue compartments, which may be masked if molecular analysis is not appropriately stratified.

### Stromal miRNA expression profiles distinguish CRC tissue from paired normal colonic tissue

**Figure 2:**
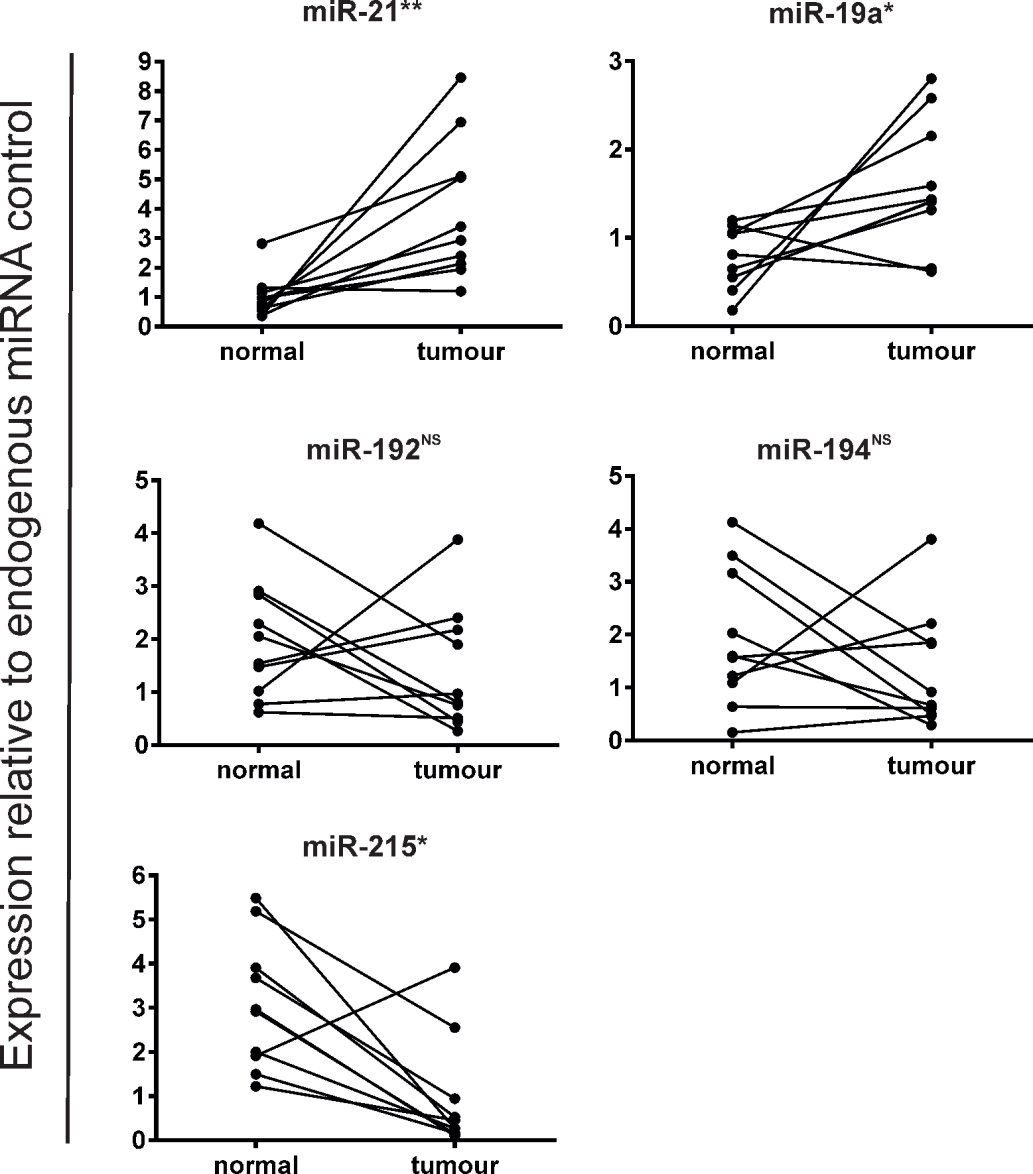
Validation of stromal miRNA candidates deregulated in fresh-frozen CRC vs. paired normal colonic tissue by Taqman®qRT-PCR (*p < 0.05; **p < 0.005; ^NS^= not significant)

To validate our findings, we examined expression of the most highly deregulated stromal miRNAs by QuantimiR™ PCR profiling using the more sensitive and specific Taqman®qRT-PCR technique in all 10 paired CRC specimens. Mean expression of miR-21 and miR-19a was X4.0 and X2.1 fold greater in tumor stroma compared with paired normal stroma (*p* < 0.05). MiR-192 and miR-194 were not significantly differentially expressed, however, a X3.3 fold reduction in miR-215 expression in tumor tissue did reach statistical significance (*p* < 0.05) (Figure 2).

These data suggest that deregulated stromal miRNAs may be capable of distinguishing CRC tissue from normal colonic tissue and support the notion that the response of the tumor microenvironment during malignant transformation is dynamic.

### Stromal miRNA expression profiles distinguish metastatic from non-metastatic CRC specimens

To identify stromal miRNA candidates with specific relevance during CRC progression we reanalyzed our QuantimiR™ qPCR data to characterize differences in miRNA expression between the stroma of early stage (Duke's A) (*n* = 5) and late stage (Duke's C) (*n* = 5) CRC specimens.

**Figure 3:**
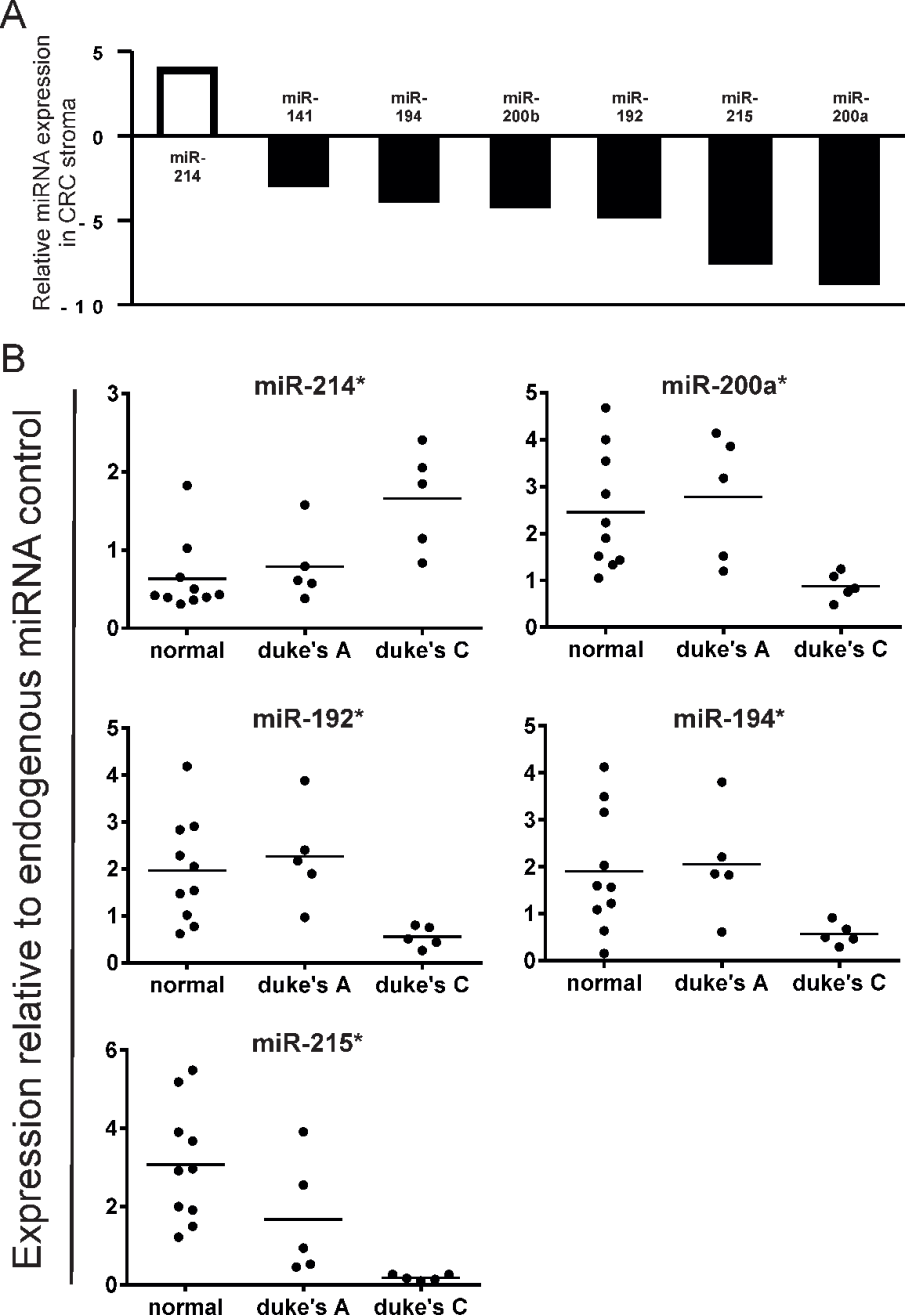
(A) Comparison of stromal miRNA expression in late stage (Duke's C) vs. early stage (Duke's A) CRC by QuantimiR™-qPCR MiRNA candidates displayed are differentially expressed by a factor > 2 (* p* < 0.05). **(B)** Validation of stromal miRNA candidates differentially expressed in late vs. early stage CRC by Taqman®qRT-PCR (*p < 0.05).

Of the 95 miRNAs examined, 7 were found to be significantly differentially expressed by a factor > 2 between Duke's A and Duke's C tumors (Figure 3A). Taqman® validation confirmed that mean miR-214 expression was increased X2.1 fold in Duke's C specimens compared with Duke's A (*p* < 0.05), whereas miR-192 and mir-194 expression were relatively suppressed by a factor of X4.1 and X3.6 respectively (*p* < 0.05) (Figure 3B).

The most highly suppressed stromal miRNAs in Duke's C compared with Duke's A tumors by QuantimiR™ (miR-200a and miR-215), were also shown to be suppressed by Taqman® qPCR, (*p* < 0.05) (Figure 3B).

These data, which identify distinct patterns of stromal miRNA expression in metastatic and non-metastatic tumor groups, highlight the potential prognostic and diagnostic applications of stromal non-coding RNA molecules in CRC.

### Robust miRNA profiles are extracted from formalin-fixed paraffin-embedded and fresh-frozen CRC tissue archives

Next, we wanted to examine the utility of stromal miRNA expression profiling in a ‘real-world’ clinical scenario.

**Figure 4:**
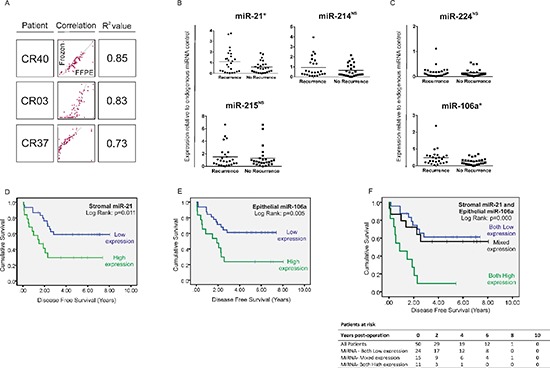
(A) Correlation between the expression status of 95 cancer relevant miRNAs in fresh-frozen and FFPE tissue by QuantimiR™-qPCR, from 3 CRC specimens chosen at random from our archive Expression of candidate stromal and epithelial miRNAs in stage II CRC specimens with (*n* = 25) and without (*n* = 25) disease recurrence at 5 years. Expression of **(B)** stromal and **(C)** epithelial miRNA candidates by Taqman®qPCR from total RNA extracted from FFPE LMD tissue. Stromal miR-21 expression and epithelial miR-106a expression were 1.84-fold and 1.88-fold greater respectively, in tumors which later metastasize compared with those which do not (*p < 0.05; ^NS^ = not significant). Kaplan-Meier curves of disease-free survival in patients with stage II disease based on significantly differentially expressed stromal and epithelial miRNAs. Patients were stratified into high or low expression groups based around group mean stromal miR-21 expression **(D)** or group mean epithelial miR-106a expression **(E)**; or expression of stromal miR-21 and epithelial miR-106a in combination **(F)** Stromal miR-214 and miR-215 and epithelial miR-224 were ubiquitously expressed between groups and were therefore not included in survival analysis (B and C).

In the current study we used FFPE tissue, as most centers in the UK performing surgery for CRC preferentially use this method of tissue preservation. However, in terms of the quality and stability of RNA in long term storage, flash-freezing in liquid nitrogen has traditionally been considered a superior technique. Therefore to ensure our approach was adequate, QuantimiR™ qPCR miRNA microarrays were used to compare expression of 95 biologically relevant miRNAs in paired FFPE and fresh-frozen tissue in a representative number (*n* = 3) of CRC specimens selected at random from our archive (Figure 4A).

We found that miRNA expression correlated closely in CRC tissue preserved by the two techniques (R^2^=0.73–0.89) (Figure 4A). These data support an increasing body of evidence which suggests that miRNAs are stable over time in archived FFPE tissue and that robust molecular profiles may be generated from CRC specimens fixed in formalin as well as fresh-frozen material. [27, 28]

### Stromal and epithelial miRNAs predict patterns of tumor recurrence in stage II CRC

To examine the prognostic utility of stromal miRNA expression profiling in stage II (lymph node negative, metastasis negative) CRC, a database of patients was generated from our extensive archive of FFPE tumor tissue. Of this carefully matched group of patients with no histological or clinical features of biologically aggressive disease, half (*n* = 25) subsequently developed metastases within 5 years and half remained metastasis free (Table 1). Median follow-up for this cohort of 50 patients was 6.08 years (95% CI: 4.98–7.19).

**Table 1:** Demographic data for all patients with stage II CRC with (stage II-R) and without (stage II-NR) metastasis at 5 years

Characteristics	Stage II-R (*n* = 25)	Stage II-NR (*n* = 25)	*p* value
Age, years at diagnosis (mean+sd)	73.92 (11.45)	71.36 (11.57)	0.435^a^
Gender (m:f) absolute numbers	21:4	17:8	0.321^b^
Tumour site			0.482^b^
Right Colon	6	9	
Left Colon	11	12	
Rectum	7	4	
Stoma	1	0	
Surgical setting			0.762^c^
Elective	18	16	
Emergency	7	9	
Histopathological data			
Maximum tumour diameter (mm)Mean (SD)	50.22 (26.00)	49.54 (18.70)	0.919^a^
T stage			0.347^b^
T2	0	2	
T3	18	16	
T4	7	7	
N stage			
N0	25	25	
M stage			
M0	25	25	
AJCC staging 7^th^ ed			0.347^b^
Stage I	0	2	
Stage IIA	18	16	
Stage IIB	7	7	
R0 resection margin	22	23	1.000^c^
Differentiation status			0.177^b^
Poor	2	2	
Moderate-Poor	4	1	
Moderate	7	14	
Well-Moderate	12	8	
Well	0	0	
Extramural vascular invasion	4	1	0.349^c^
Tumour perforation	1	2	1.000^c^
Adjuvant therapy			
Neoadjuvant Chemotherapy	3	2	1.000^c^
Neoadjuvant Radiotherapy	4	2	0.667^c^
Adjuvant Chemotherapy	6	2	0.247^c^
Adjuvant Radiotherapy	1	2	1.000^c^
Mean follow up, years (s.d)	2.89 (1.62)	5.40 (2.02)	0.000^a^

a = Independent *t*-test,

b = Chi squared test,

c = Fisher's exact test

Each CRC specimen was subjected to LMD and total RNA was extracted separately from tumor stroma and epithelium. Expression of miRNA candidates identified previously (Figure 2), were subsequently examined by Taqman®qRT-PCR.

In stroma, mean miR-21 expression was X1.84-fold greater in stage II tumors from patients who developed metastatic recurrence (stage II-R) compared with patients who did not (stage II-NR) (*p* < 0.05) (Figure 4B). Stratifying patients into high and low expression groups relative to mean miR-21 expression for the group as a whole (mean = 0.857; 95% CI: 0.607–1.107) revealed that 13 out of 25 patients with stage II-R disease expressed high levels of stromal miR-21 compared with 6 out of 25 patients with stage II-NR disease (*p* < 0.05).

Patients expressing high levels of stromal miR-21 had significantly shorter disease free survival (DFS) (HR = 2.68, 95% CI: 1.21–5.93, *p* = 0.015) (Figure 4D) and overall survival (OS) (HR = 2.47, 95% CI:1.19–5.55, *p* = 0.029) (Figure 6A) than patients expressing low levels of stromal miR-21. 13 out of 19 patients with high stromal miR-21 expression developed distant metastases during the period of follow-up compared with 12 out of 31 patients in the low stromal miR-21 expression group (*p* < 0.05).

Stromal miR-214 and miR-215 were not significantly differentially expressed in recurrence and non-recurrence groups and so were not included in survival analysis (Figure 4B). However, we did want to examine whether panels of deregulated miRNA expression provided better prognostic discrimination than miRNA candidates examined in isolation, which prompted us to extend our analysis to epithelial miRNAs identified in our QuantimiR™ screen (Figure 1B).

Mean expression of miR-224, the most upregulated miRNA candidate identified in CRC epithelium, was not significantly different in LMD epithelium from stage II-R and stage II-NR tumor specimens (Figure 4C). However, mean expression of miR-106a, the second most deregulated epithelial miRNA was increased X1.88 fold in the LMD epithelium of stage II-R CRC specimens compared with stage II-NR specimens (*p* < 0.05) (Figure 4C). When patients were stratified into high and low miR-106a expression groups (mean = 0.372; 95% CI: 0.265–0.478), patients expressing high levels of epithelial miR-106a had significantly shorter DFS (HR = 2.91, 95% CI: 1.32–6.42, *p* = 0.008) (Figure 4E) and OS (HR = 2.25, 95% CI: 1.00–5.04, *p* = 0.049) (Figure 6B) than patients expressing low levels of epithelial miR-106a. 13 out of 18 patients with high epithelial miR-106a expression developed distant metastases during the period of follow-up compared with 12 out of 32 patients in the low epithelial miR-106a expression group (*p* < 0.05).

When considered in combination, high expression of stromal miR-21 and epithelial miR-106a were also associated with poor DFS (Both High vs. Both Low: HR = 5.09, 95% CI: 2.02–12.85, *p* = 0.001) (Figure 4F) and OS (Both High vs. Both Low: HR = 4.13, 95% CI: 1.48–11.52, *p* = 0.007) (Figure 6D).

These data suggest that panels of deregulated miRNA expression from FFPE tissue, a widely available clinical resource, may provide practical prognostic information for clinicians treating patients with CRC.

### MiRnome-wide miRNA profiling of laser microdissected tumor tissue reveals further promising prognostic bio-markers in CRC

Prompted by the successful combination of high-throughput molecular screening and LMD, we broadened our approach to encompass miRnome-wide gene expression analysis.

From our cohort of 50 patients with stage II disease, 10 stage II-R and 10 stage II-NR FFPE CRC specimens were selected at random (Table 2). Each was subjected to LMD and total-RNA was extracted separately from epithelial and stromal tumor strata. Global epithelial and stromal miRNA expression profiling was conducted using the 7^th^ generation miRCURY LNA™ high-throughput screening platform.

**Table 2:** Demographic data for patients selected at random from our archive to examine for array-based differential gene expression between stage II CRC with (stage II-R) and without (stage II-NR) recurrence at 5 years

Characteristics	Stage II-R (*n* = 10)	Stage II-NR (*n* = 10)	*p* value
Age, years at diagnosis (mean+sd)	73.90 (11.31)	76.20 (8.43)	0.612^a^
Gender (m:f) absolute numbers	8:2	6:4	0.329^b^
Tumour site			0.132^b^
Right Colon	2	5	
Left Colon	2	4	
Rectum	5	1	
Stoma	1	0	
Surgical setting			0.370^c^
Elective	4	7	
Emergency	6	3	
Histopathological data			
Maximum tumour diameter (mm)Mean (SD)	50.50 (26.71)	49.50 (10.12)	0.913^a^
T stage			0.171^b^
T2	0	2	
T3	10	7	
T4	0	1	
N stage			
N0	10	10	
M stage			
M0	10	10	
AJCC staging 7^th^ ed			0.171^b^
Stage I	0	2	
Stage IIA	10	7	
Stage IIB	0	1	
R0 resection margin	10	9	1.000^c^
Differentiation status			0.306^b^
Poor	0	1	
Moderate-Poor	2	1	
Moderate	3	6	
Well-Moderate	5	2	
Well	0	0	
Extramural vascular invasion	1	0	1.000^c^
Tumour perforation	0	0	
Adjuvant therapy			
Neoadjuvant Chemotherapy	0	0	
Neoadjuvant Radiotherapy	0	0	
Adjuvant Chemotherapy	0	0	
Adjuvant Radiotherapy	0	0	
Mean follow up, years (s.d)	3.37 (1.41)	5.90 (2.23)	0.007^a^

a = Independent *t*-test,

b = Chi squared test,

c = Fisher's exact test

**Figure 5:**
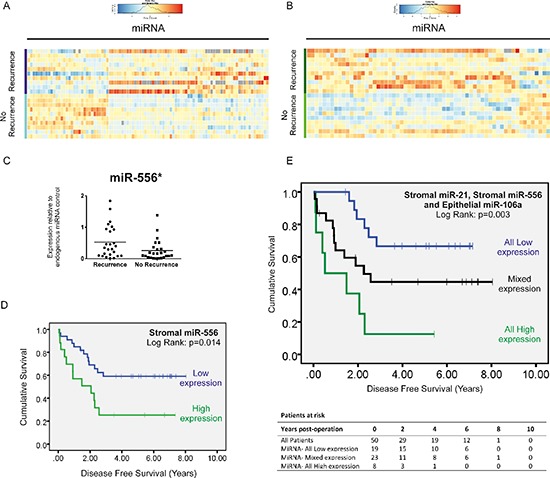
MiRnome-wide, high through-put miRNA screening: Heatmap of miRNAs differentially expressed (adjusted *p* < 0.05) in LMD stroma (A) and epithelium (B) between stage II CRC specimens with and without recurrence within 5 years **(C)** Taqman®qPCR validation reveals that miR-556 the most highly deregulated miRNA in our screen, is overexpressed x2.02-fold in the stroma of stage II CRC specimens which later metastasize compared with those that do not. **(D)** Kaplan-Meier curve of disease free survival in patients with stage II disease. Patients were stratified into high or low expression groups based around mean stromal miR-556 expression for the group as a whole. **(E)** Kaplan-Meier curve of disease free survival in patients with stage II disease. Patients were stratified into high or low expression groups based around mean stromal miR-21 and miR-556 and epithelial miR-106a expression for the group as a whole, and subdivided into groups expressing all candidate miRNAs at low levels, one or two candidate miRNAs at low levels or all miRNA candidates at high levels. (*p < 0.05)

In total, 95 miRNAs were significantly differentially expressed in stage II-R CRC stroma compared with stage II-NR stroma (Figure 5A), of which 10 were up- or down-regulated by a factor > 2 (*p* < 0.05). In contrast, 63 epithelial miRNAs were significantly differentially expressed (Figure 5B); 6 by a factor > 2 (*p* < 0.05).

**Figure 6:**
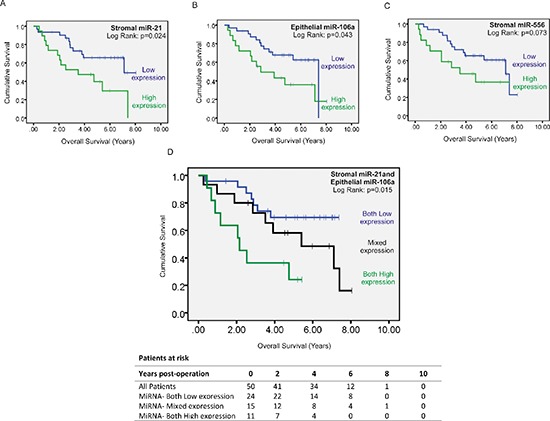
Kaplan-Meier curve of overall survival in patients with stage II disease Patients were stratified into high or low expression groups based around group mean stromal miR-21 expression **(A)** group mean epithelial miR-106a expression **(B)** and group mean stromal miR-556 expression **(C)** As elevated stromal miR-21 and epithelial miR-106a expression predicted significantly reduced overall survival they were included in combined analysis **(D)** whereas stromal miR-556 expression was excluded.

Subsequently we validated expression of our top scoring stromal candidate, miR-556, by conducting Taqman® qRT-PCR analysis in all 50 LMD tumor specimens. This confirmed mean miR-556 expression is increased X2.02-fold in the stroma of stage II CRC specimens which go on to metastasize compared with those which do not (*p* < 0.05) (Figure 5C).

When we stratified patients into high and low expression groups relative to mean expression (mean = 0.393; 95% CI: 0.266–0.519), those expressing high levels of stromal miR-556 had significantly shorter DFS (HR = 2.60, 95% CI: 1.18–5.73, *p* = 0.018) (Figure 5D), but not significantly shorter OS (Figure 6C) than patients expressing low levels of stromal miR-556. 12 out of 17 patients with high stromal miR-556 expression developed distant metastases during the period of follow-up compared with 13 out of 33 patients in the low stromal miR-556 expression group (*p* < 0.05).

When all three potentially prognostically relevant miRNA candidates identified in this study were considered in combination (stromal miR-21 and miR-556; and epithelial miR-106a), we found that metastases developed in 7 out of 8 patients expressing high levels of all three candidates, compared with 12 out of 23 patients expressing high levels of two or one candidate; and 6 out of 19 patients expressing none of the candidates at high levels (*p* < 0.05). Furthermore, those expressing high levels of all three miRNAs had significantly shorter DFS (All High vs. All Low: HR = 5.83, 95% CI: 1.92–17.57; *p* = 0.002) (Figure 5E) (Patient characteristics may be found in Table 3).

**Table 3:** Demographic data for patients with stage II CRC stratified according to expression of stromal miR-21, stromal miR-556 and epithelial miR-106a in tumor

Characteristics	All Low (*n* = 19)	High/Low mix (*n* = 23)	All High (*n* = 8)	*p* value
Age, years at diagnosis (mean+sd)	71.58 (13.52)	74.09 (10.17)	71.00 (10.57)	0.715^a^
Gender (m:f) absolute numbers	13:6	18:5	7:1	0.537^b^
Tumour site				0.730^c^
Right Colon	6	8	1	
Left Colon	10	9	4	
Rectum	3	5	3	
Stoma	0	1	0	
Surgical setting				0.285^b^
Elective	12	18	4	
Emergency	7	5	4	
Histopathological data				
Maximum tumour diameter (mm) Mean (SD)	53.37 (21.94)	50.00 (25.91)	41.25 (9.10)	0.445^a^
T stage				0.470^c^
T2	1	1	0	
T3	14	16	4	
T4	4	6	4	
AJCC staging 7^th^ ed				0.470^c^
Stage I	1	1	0	
Stage IIA	14	16	4	
Stage IIB	4	6	4	
R0 resection margin	18	20	7	0.803^b^
Differentiation status				0.347^c^
Poor	2	1	1	
Moderate-Poor	2	1	2	
Moderate	10	9	2	
Well-Moderate	5	12	3	
Well	0	0	0	
Extramural vascular invasion	2	1	2	0.248^b^
Tumour perforation	1	2	0	0.662^b^
Adjuvant therapy				
Neoadjuvant Chemotherapy	3	1	1	0.454^b^
Neoadjuvant Radiotherapy	3	2	1	0.780^b^
Adjuvant Chemotherapy	2	3	3	0.190^b^
Adjuvant Radiotherapy	2	0	1	0.252^b^
Mean follow up, years (s.d)	4.76 (1.74)	4.02 (2.49)	3.05 (2.11)	0.176^a^

a = One way ANOVA,

b = Chi squared test,

c = Kruskal-Wallace

## DISCUSSION

Understanding the molecular events which underpin the metastatic cascade is important not only for the development of novel drugs, but also because categorizing heterogeneous tumors based on their molecular characteristics may lead to enhanced prognostication and better tailored treatment.

This is particularly relevant for patients with stage II CRC. Disease recurrence remains a substantial problem in this group as 20–25% of patients following surgery with curative intent go on to develop metastases within 5 years [1]. Although this clearly represents a significant minority of patients, the blanket use of adjuvant treatment is not supported by evidence in this group [29]. Thus a molecular staging approach enabling early identification of patients at high risk of tumor recurrence would create the opportunity both to provide targeted interventions where necessary, and avoid overtreatment in other cases.

MiRNAs, which are non-coding RNA molecules with promising clinical applications, [30, 31] are well suited to this task. The analysis of miR-21 in plasma and stool [32–34] for example, has been identified as a potential diagnostic/screening tool in CRC and high miR-21 expression in tumor [14] and serum [35] predicts poor post-operative outcomes.

MiRNAs also play significant roles during tumor initiation and progression, and an important theme in recent years is the emergence of pro-metastatic events in the tumor microenvironment regulated by miRNAs [16].

In the present study, miR-21 was the most highly upregulated stromal miRNA candidate in CRC tissue compared with paired ‘normal' stroma by Taqman®qRT-PCR. In a separate analysis of 50 patients with stage II CRC, stromal miR-21 was also significantly overexpressed in patients who developed metastases within 5 years compared with those that remained metastasis free.

MiR-21 is an important oncogene; antisense inhibition of miR-21 induces cellular apoptosis in cooperation with EGFR-tyrosine kinase inhibitors [36] and enhances sensitivity to radiotherapy and chemotherapy in a number of tumor contexts [37–39]. MiR-21 upregulation also enhances proliferation and invasion in pancreatic, glioblastoma and CRC cell lines [40–42]. However to date, all chemo-resistance and pro-metastatic mechanisms described have been limited to the malignant epithelium and yet observations from the current study contribute to a growing body of evidence which suggests deregulation of miR-21 is a stromal phenomenon in CRC, and not just a feature of cancer cells [15, 20, 43].

MiR-106a is also considered oncogenic in CRC. In contrast to miR-21, miR-106a was identified exclusively in LMD CRC epithelium in this study. MiR-106a is highly expressed in serum and primary malignant tissue of patients with CRC, [14, 44] and its presence in feces from tumor cell exfoliation into the gastrointestinal tract, has been identified as a potential adjuvant screening tool for colonic malignancies [45]. MiR-106a expression in CRC cell lines is also associated with enhanced invasion *in vitro* and *in vivo* and has been linked to known pro-metastatic molecular pathways through direct translational suppression of transforming growth factorβ receptor 2 (TGFBR2) [46].

Intriguingly, both miR-21 and miR-106a were identified in a previous study which compared miRNA expression in CRC and paired normal colonic tissue. However, in contrast to Schetter et al., [14] who used whole tumor tissue sections, we performed an initial LMD step prior to profiling with high-throughput microarrays. Our approach, which was designed to avoid masking important biological differences between cancer cells and their supportive microenvironment, also allows us to map potentially relevant molecular events to the appropriate tumor compartment.

Crucially, we found that both elevated stromal miR-21 and epithelial miR-106a were associated with significantly reduced DFS and OS in patients with stage II CRC, which encouraged further interrogation of our QuantimiR™ screen:

MiR-215, miR-192 and miR-194 were each downregulated more than X2-fold in CRC stroma compared with paired ‘normal' stroma, but only miR-215 suppression was validated successfully by Taqman®.

Collective downregulation of all 3 miRNAs has previously been identified in nephroblastoma and is associated with upregulated activin receptor type 2B (ACVR2B) expression in the TGFβ pathway [47]. MiR-192 and mir-215 are also positively regulated by the tumor suppressor p53 and are capable of inducing cell cycle arrest and regulating cellular adhesion and proliferative functions in a p53/p21 dependent manner [48]. Furthermore, miR-215 suppression has been demonstrated in CRC, in a study which paradoxically linked declining miR-215 expression with improved overall survival [49]. However, as patterns of stromal miRNA expression are potentially made less distinct by the presence of extraneous epithelial tissue, this paradox may be explained by the use of bulk tumor specimens rather than LMD tissue.

In the present study, the only stromal miRNA upregulated more than 2X-fold in late stage (Duke's C) vs. early stage (Duke's A) CRC specimens, was miR-214. MiR-214 plays a key role in tissue fibrosis, a pathological process driven from the stroma [50, 51] and furthermore, upregulated stromal miR-214 expression was described previously in CRC in a study which compared LMD tumors with unpaired normal colonic tissue [21]. It is reassuring that several of our observations are mirrored in this complementary study by Nishida et al., namely that miR-214, miR-21 and members of the miR-17–92a cluster are strongly upregulated; and miR-215, miR-192 and miR-194 downregulated in CRC stroma. However, having identified miRNA candidates deregulated in CRC stroma, the authors did not ascertain their prognostic significance, nor did they examine differentially expressed miRNAs in tumor vs. normal epithelium [21].

Here, the utility of deregulated miRNAs such as miR-21 in CRC stroma and miR-106a in CRC epithelium, were scrutinized in stage II disease, in a ‘real-world’ clinical scenario which presents significant prognostic and therapeutic challenges. In contrast, we found that stromal miR-214 and miR-215 were ubiquitously expressed in all stage II CRC specimens regardless of metastatic status at 5 years and hence they were not included in survival analysis. Nevertheless, miR-214 and miR-215 may still be biologically relevant in CRC and this raises an interesting point; that the same miRNAs deregulated in cancer are not necessarily deregulated during metastatic progression. Crucially, this observation supports the view that molecular mechanisms underpinning the metastatic cascade are distinct from those which drive carcinogenesis [52].

To broaden and enrich our search for clinically relevant miRNAs, we adopted a miRnome-wide profiling approach to specifically compare miRNA expression in stage II CRC specimens with and without recurrence at 5 years. In this comparison of 20 patients (10 stage IIR vs.10 stage IINR) 95 consistently deregulated miRNAs were identified in stroma compared with 63 miRNAs in tumor epithelium.

Most cancer research is focused on tumor cells, but because stromal cells are less likely to acquire *de-novo* mutations, evade anti-cancer immunity, or develop drug resistance than malignant epithelial cells, the concept of stroma targeted therapy has become increasingly attractive [53, 54]. Another important theme in recent years is the development of therapies which target or exploit non-coding RNAs [30, 31, 34]. In this context, the characterization of miRNAs differentially expressed in metastatic vs. non-metastatic tumor groups may reveal opportunities to develop novel pharmacological interventions both in malignant tissue and the supportive tumor microenvironment.

MiRNAs such as miR-21 and miR-320 which are known to be active in the tumor microenvironment, have important regulatory roles within stromal cell such as fibroblasts, and in the malignant state impact on disease progression by modulating cellular secretory functions or facilitating other changes in cellular phenotype [15, 17, 20]. Other miRNAs are selectively sequestered into exosomes and actively exported from cells in a manner which is dependent both on gatekeeper proteins and specific miRNA sequence motifs. [55] Crucially, exosomal miRNA transfer from stromal cells to target cells also impacts significantly on disease progression in various tumor contexts [56, 57].

In the current study miR-556 was the most upregulated stromal miRNA identified in stage II-R CRC specimens. To establish whether stromal miR-556 has a significant biological role during the metastatic cascade will require further detailed in silico, functional and mechanistic studies. However, regardless of any putative biological function, deregulated stromal miR-556 may have a role as a biomarker of disease progression in CRC as the current study demonstrates an association between stromal miR-556 expression and decreased DFS stage II disease. In particular, when combined with stromal miR-21 and epithelial miR-106a, stromal miR-556 contributed to a distinct molecular signature of disease recurrence: At 3 years following surgery with curative intent, 3/19 (16%) patients expressing low levels of all three miRNAs had developed metastases compared with 10/23 (43%) patients expressing high levels of one or two miRNAs and 7/8 (88%) patients expressing high levels of all three miRNAs.

In summary, we have demonstrated that stromal and epithelial miRNA profiles may be used to identify patients at high risk of CRC recurrence and reduced OS. Our data supports the notion that miRNAs are important actors during the metastatic cascade, and that miRNA profiling may be a valuable aid to therapeutic decision making.

Furthermore, by demonstrating that miRNA profiles from fresh frozen CRC tissue correlate closely with FFPE tissue, a widely available clinical resource, we highlight the translational appeal of this research. However, in order to fully evaluate these findings, additional validation in a larger and independent cohort will be required.

## MATERIALS AND METHODS

### Clinical samples

Consecutive pairs of primary colonic tumor and adjacent non-tumorous colonic tissue were obtained during surgery at University Hospital Southampton as part of a prospective National Institute of Health Research study (UKCRN ID 6067). Although secreted miRNAs are present within the tumor microenvironment [58, 59] and their paracrine effects are not yet fully understood, [60] for the purpose of this study, macroscopically normal tissue > 5 cm from tumor margin was assumed to be non-malignant. This is consistent with traditional surgical practice which requires more than 5cm of oncological clearance in order to ensure microscopic mesenteric lymph node deposits are not excluded from the resection specimen [61, 62].

Half of each specimen was frozen in liquid nitrogen and stored at −80°C, and half fixed in formalin and embedded in paraffin. From this archive, 5 early stage (Duke's A) and 5 late stage (Duke's C) specimens were selected at random to examine for array-based differential gene expression between normal and tumor tissue.

Subsequently, a further 50 consecutive and formalin fixed paraffin embedded (FFPE) CRC specimens were obtained from the study cohort. This cohort comprised 25 patients with stage II disease who developed metastasis within 5 years and 25 carefully matched patients who remained metastasis free in that time (Table 1). Pathological verification of diagnosis and staging was in accordance with the Association of Coloproctology of Great Britain and Ireland guidelines on the management of CRC [63].

All specimens were stored in a designated UK Human Tissue Act approved tumor bank. Comprehensive demographic, clinical and pathological information was collated and hereditary tumors, mucinous tumors, and tumors with histologically identified necrosis excluded. All patients provided written informed consent and regional ethical approval was obtained.

### Slide preparation, laser capture microdissection and RNA extraction

Frozen specimens were sectioned using a cryostat at 8–10 μm thickness onto membrane-mounted slides (Molecular Devices). FFPE specimens were sectioned using a microtome and subjected to deparaffinisation in Xylene for 1 minute. Tissue sections were then fixed in 75% ethanol for 30s and stained with Cresyl Violet for 1 min before undergoing a further dehydration step in 100% ethanol. Once air dried, LMD was performed using the Leica AS LMD microdissection platform (Leica Microsystems, UK) and cut tissue was collected directly into 50 μl of cell lysis buffer (Applied Biosystems). Approximately 1 × 10^6^ μm^2^ was dissected from each slide. Malignant colonic epithelium and tumor associated stroma and normal colonic epithelium and normal stroma were each microdissected separately.

Total RNA extraction was conducted immediately using either the RNAqueous®-Micro Kit (Ambion) for fresh-frozen tissue or the RecoverAll™ kit (Ambion) for FFPE in accordance with the manufacturer's instructions. RNA concentration and quality was determined using the Nanodrop™ 1000 spectrophotometer (Thermo Scientific) and a ratio of absorbance at 260 nm and 280 nm ≈ 2 was considered ‘pure'.

### RT-PCR for cancer array plate with QuantimiR^TM^

The Cancer Array plate with QuantiMiR^TM^ (Cambridge Biosciences) utilizes a reverse transcription (RT) step to convert miRNAs into quantifiable cDNAs which are then amplified for 95 different cancer-associated miRNAs and detected using SYBR Green fluorescent dye. This kit was used according to the manufacturer's instructions. Briefly, 100 ng total RNA was dried down by vacuum centrifugation and resuspended in 5 μl of nuclease-free water. The RNA was modified by the addition of a polyA tail to which an oligo dT adaptor was annealed. The RT reagents from the kit (5 × buffer, dNTP mix, DTT, RNase-free dH_2_O and reverse transcriptase enzyme) were added to the modified RNA and the RT step was performed at 42°C for 60 min then inactivated at 95°C for 10 min. cDNA was mixed with 2 × Power SYBR® master mix (Applied Biosystems), RNase-free dH_2_O and a universal reverse primer (complementary to the adaptor sequence on each cDNA molecule) and the mix was aliquoted into 96 wells of an optical microplate. 95 forward primers (10 μmoles) specific to a selection of cancer-related miRNAs and one for the U6 small RNA control were added to separate wells on the plate. cDNA was amplified using the ABI-HT7500 qPCR instrument (Applied Biosystems) and the following cycling parameters (50°C for 2 min; 95°C for 10 min and 40 cycles of 95°C for 15 sec and 60°C for 1 min). Expression levels, normalized with the global median value for the whole plate, were calculated using the ΔΔC_T_ method and expressed relative to one of the specimens that was assigned the value 1.

### MiRnome wide gene expression analysis

Microarray data acquisition and presentation was conducted in accordance with MIAME guidelines [64]. The biomarker potential of data acquired was assessed in accordance with REMARK guidelines [65].

Briefly, 100 ng total RNA was dried down by vacuum centrifugation and resuspended in 3 μl of nuclease-free water. Hy3™ fluorescence labelled RNA was prepared using the miRCURY LNA™ microRNA Array Hi-Power labelling kit (Exiqon) in accordance with the manufacturer's instructions. For each labelling reaction, 3 μl total RNA was combined with 1 μl ‘Spike-in' miRNA control, 0.5 μl CIP buffer and 0.5 μl CIP enzyme. The ‘Spike-in' control contains 52 different synthetic unlabeled miRNAs corresponding to capture probes on the miRCURY LNA™ 7^th^ generation microRNA microarrays from Exiqon and various concentrations. This reaction mix was incubated for 30 minutes at 37°C using a PCR cycler with heated lid. After 30 minutes, the enzyme reaction was stopped and the RNA denatured by incubation at 90°C followed by snap cooling on ice for 5–15 minutes. Subsequently this 5 μl reaction mix was combined with 1.5 μl Hy3™ fluorescent label, 3 μl Hi-Power labelling buffer, 2 μl DMSO and 1 μl of Hi-Power labelling enzyme and incubated in darkness at 16°C. To stop the Hi-Power labelling reaction after 2 hours the sample was heated to 65°C for 15 minutes and then placed immediately on ice.

Labelled RNA was subsequently hybridized to miRCURY LNA™ 7^th^ generation microRNA microarrays using a commercially available kit from Exiqon. Hybridization was conducted for 16 hours at 56°C following which, array slides were washed, dried by cerntifugation and scanned using a GenePix 4000B scanner (Molecular Devices) to detect Hy3™ at a wavelength of 532 nm. Image analysis was performed using GenePixPro 3.0.5 software, which permitted a miRbase v19 updated gal file (www.exiqon.com/Gal-downloads) containing a mapping grid to be superimposed and accurately aligned onto the array image in TIFF format.

Whole miRnome microarray data was processed by subtracting background signals from the foreground signals for each feature. Any features which had a non-zero flag or a background subtracted signal < 0.5 was set to ‘missing.' Expression signals were then log2 transformed and quartile normalized. After normalisation, any features targeting non-human miRNAs were removed. Data was sorted according to fold change (> 2) and *p*-value (< 0.05).

The nature of the platform meant that each miRNA probe was represented 4 times. For each miRNA, the mean value from 4 data points was used and an expression ratio calculated between LMD stroma/epithelium from recurrence and non-recurrence groups.

### Quantitative expression analysis using Taqman^®^ qRT-PCR

For each qPCR assay 2.5 ng total RNA from LMD samples (0.5 ng/μl) was converted into cDNA using a miRNA-specific RT step. The TaqMan® MicroRNA Reverse Transcription kit (Applied Biosystems) was used according to the manufacturer's instructions in conjunction with a miRNA-specific looped reverse primer. The RT reagents (10 × RT buffer, dNTPs, RNase Inhibitor, RNase-free dH_2_O and MultiScribe^TM^ Reverse transcriptase (50 U/μl)) were combined with total RNA and the appropriate RT primer was added to the tube. The following RT reaction conditions were applied; 16°C for 30 min; 42°C for 30 min, 85°C for 5 min and 4°C hold. For the 20 μl PCR reaction; 10 μl Taqman 2 × Universal PCR Master Mix (Applied Biosystems) was added to 1.33 μl of cDNA, 7.67 μl RNase-free dH_2_O and 1 μl of Taqman MicroRNA Assay primer and probe mix. Taqman Primer and probe mixes supplied by Applied Biosystems, used in the study include U6B (4427975; 001093), hsa-miR-224 (4427975; 000599), hsa-miR-214 (4427975; 000517), hsa-miR-215 (4427975; 000518), hsa-miR-21 (4427975; 00397), hsa-miR-19a (4427975; 002424), hsa-miR-192 (4427975; 000491), hsa-miR-194 (4427975; 000493), hsa-miR-200a (4427975; 001011), hsa-miR-556 (4427975; 002344) and hsa-miR-106a (4427975; 000578).

Reactions were performed in triplicate in 96 well plates covered with optical adhesive seal. The ABI-HT7500 qPCR instrument (Applied Biosystems) was used with the following cycling parameters: 95°C for 10 min and 40 cycles of 95°C for 15 sec and 60°C for 60 sec. Expression levels, normalized with U6 qPCR assay were calculated using the ΔΔC_T_ method and expressed relative to one of the specimens that was assigned the value 1.

### Statistical analysis

Differences between paired tumor and normal tissue samples were expressed as fold-change, using the mean expression value from the normal group as the reference and the Wilcoxon signed rank test for statistical significance. Differences between Duke's A and Duke's C tumor groups were expressed as fold-change using the mean value from the Duke's A group as a reference and the Mann-Whitney *U* test for statistical significance. Differences between recurrence and non-recurrence groups in stage II disease were expressed as fold-change using the mean value from the non-recurrence group as a reference and an unpaired student's *t*-test for statistical significance. The threshold level of significance was set at 0.05 for all tests.

Correlation analysis between FFPE and fresh-frozen tissue, using QuantimiR™ miRNA array data was conducted by normalizing CT values using the “norm.rankinvariant” method from the R Bioconductor package “HTqPCR”: http://www.bioconductor.org/packages/release/bioc/html/HTqPCR.html

Data is presented in a scatterplot format. Pearson correlation coefficients are reported.

## References

[1] Manfredi S, Bouvier AM, Lepage C, Hatem C, Dancourt V, Faivre J (2006). Incidence and patterns of recurrence after resection for cure of colonic cancer in a well defined population. The British journal of surgery.

[2] Ferlay J, Parkin DM, Steliarova-Foucher E (2010). Estimates of cancer incidence and mortality in Europe in 2008. Eur J Cancer.

[3] van der Pool AEM, Damhuis RA, Ijzermans JNM, de Wilt JHW, Eggermont AMM, Kranse R, Verhoef C (2012). Trends in incidence, treatment and survival of patients with stage IV colorectal cancer: a population-based series. Colorectal Disease.

[4] Simmonds PC (2000). Palliative chemotherapy for advanced colorectal cancer: systematic review and meta-analysis. Colorectal Cancer Collaborative Group. BMJ.

[5] Grothey A, Sargent D, Goldberg RM, Schmoll HJ (2004). Survival of patients with advanced colorectal cancer improves with the availability of fluorouracil-leucovorin, irinotecan, and oxaliplatin in the course of treatment. Journal of clinical oncology: official journal of the American Society of Clinical Oncology.

[6] Cunningham D, Humblet Y, Siena S, Khayat D, Bleiberg H, Santoro A, Bets D, Mueser M, Harstrick A, Verslype C, Chau I, Van Cutsem E (2004). Cetuximab monotherapy and cetuximab plus irinotecan in irinotecan-refractory metastatic colorectal cancer. The New England journal of medicine.

[7] Hurwitz H, Fehrenbacher L, Novotny W, Cartwright T, Hainsworth J, Heim W, Berlin J, Baron A, Griffing S, Holmgren E, Ferrara N, Fyfe G, Rogers B (2004). Bevacizumab plus irinotecan, fluorouracil, and leucovorin for metastatic colorectal cancer. The New England journal of medicine.

[8] Mirnezami R, Nicholson J, Darzi A (2012). Preparing for precision medicine. The New England journal of medicine.

[9] Clarke SJ, Karapetis CS, Gibbs P, Pavlakis N, Desai J, Michael M, Tebbutt NC, Price TJ, Tabernero J (2013). Overview of biomarkers in metastatic colorectal cancer: tumour, blood and patient-related factors. Critical reviews in oncology/hematology.

[10] Bullock MD, Bruce A, Sreekumar R, Curtis N, Cheung T, Reading I, Primrose JN, Ottensmeier C, Packham GK, Thomas G, Mirnezami AH (2013). FOXO3 expression during colorectal cancer progression: biomarker potential reflects a tumour suppressor role. British journal of cancer.

[11] Slaby O, Svoboda M, Michalek J, Vyzula R (2009). MicroRNAs in colorectal cancer: translation of molecular biology into clinical application. Molecular cancer.

[12] Zhang L, Pickard K, Jenei V, Bullock MD, Bruce A, Mitter R, Kelly G, Paraskeva C, Strefford J, Primrose J, Thomas GJ, Packham G, Mirnezami AH (2013). miR-153 supports colorectal cancer progression via pleiotropic effects that enhance invasion and chemotherapeutic resistance. Cancer research.

[13] Winter J, Jung S, Keller S, Gregory RI, Diederichs S (2009). Many roads to maturity: microRNA biogenesis pathways and their regulation. Nature cell biology.

[14] Schetter AJ, Leung SY, Sohn JJ, Zanetti KA, Bowman ED, Yanaihara N, Yuen ST, Chan TL, Kwong DL, Au GK, Liu CG, Calin GA, Croce CM (2008). MicroRNA expression profiles associated with prognosis and therapeutic outcome in colon adenocarcinoma. JAMA: the journal of the American Medical Association.

[15] Yao Q, Cao S, Li C, Mengesha A, Kong B, Wei M (2011). Micro-RNA-21 regulates TGF-beta-induced myofibroblast differentiation by targeting PDCD4 in tumor-stroma interaction. International journal of cancer Journal international du cancer.

[16] Bronisz A, Godlewski J, Wallace JA, Merchant AS, Nowicki MO, Mathsyaraja H, Srinivasan R, Trimboli AJ, Martin CK, Li F, Yu L, Fernandez SA, Pecot T (2012). Reprogramming of the tumour microenvironment by stromal PTEN-regulated miR-320. Nature cell biology.

[17] Bullock MD, Pickard KM, Nielsen BS, Sayan AE, Jenei V, Mellone M, Mitter R, Primrose JN, Thomas GJ, Packham GK, Mirnezami AH (2013). Pleiotropic actions of miR-21 highlight the critical role of deregulated stromal microRNAs during colorectal cancer progression. Cell death & disease.

[18] Yang M, Chen J, Su F, Yu B, Lin L, Liu Y, Huang JD, Song E (2011). Microvesicles secreted by macrophages shuttle invasion-potentiating microRNAs into breast cancer cells. Molecular cancer.

[19] Melo SA, Sugimoto H, O'Connell JT, Kato N, Villanueva A, Vidal A, Qiu L, Vitkin E, Perelman LT, Melo CA, Lucci A, Ivan C, Calin GA (2014). Cancer Exosomes Perform Cell-Independent MicroRNA Biogenesis and Promote Tumorigenesis. Cancer cell.

[20] Nielsen BS, Jorgensen S, Fog JU, Sokilde R, Christensen IJ, Hansen U, Brunner N, Baker A, Moller S, Nielsen HJ (2011). High levels of microRNA-21 in the stroma of colorectal cancers predict short disease-free survival in stage II colon cancer patients. Clinical & experimental metastasis.

[21] Nishida N, Nagahara M, Sato T, Mimori K, Sudo T, Tanaka F, Shibata K, Ishii H, Sugihara K, Doki Y, Mori M (2012). Microarray analysis of colorectal cancer stromal tissue reveals upregulation of two oncogenic miRNA clusters. Clinical cancer research : an official journal of the American Association for Cancer Research.

[22] Gabriely G, Wurdinger T, Kesari S, Esau CC, Burchard J, Linsley PS, Krichevsky AM (2008). MicroRNA 21 promotes glioma invasion by targeting matrix metalloproteinase regulators. Molecular and cellular biology.

[23] Meng F, Henson R, Wehbe-Janek H, Ghoshal K, Jacob ST, Patel T (2007). MicroRNA-21 regulates expression of the PTEN tumor suppressor gene in human hepatocellular cancer. Gastroenterology.

[24] Asangani IA, Rasheed SA, Nikolova DA, Leupold JH, Colburn NH, Post S, Allgayer H (2008). MicroRNA-21 (miR-21) post-transcriptionally downregulates tumor suppressor Pdcd4 and stimulates invasion, intravasation and metastasis in colorectal cancer. Oncogene.

[25] Xu XM, Wang XB, Chen MM, Liu T, Li YX, Jia WH, Liu M, Li X, Tang H (2012). MicroRNA-19a and -19b regulate cervical carcinoma cell proliferation and invasion by targeting CUL5. Cancer letters.

[26] Kurokawa K, Tanahashi T, Iima T, Yamamoto Y, Akaike Y, Nishida K, Masuda K, Kuwano Y, Murakami Y, Fukushima M, Rokutan K (2012). Role of miR-19b and its target mRNAs in 5-fluorouracil resistance in colon cancer cells. Journal of gastroenterology.

[27] Xi Y, Nakajima G, Gavin E, Morris CG, Kudo K, Hayashi K, Ju J (2007). Systematic analysis of microRNA expression of RNA extracted from fresh frozen and formalin-fixed paraffin-embedded samples. RNA.

[28] Doleshal M, Magotra AA, Choudhury B, Cannon BD, Labourier E, Szafranska AE (2008). Evaluation and validation of total RNA extraction methods for microRNA expression analyses in formalin-fixed, paraffin-embedded tissues. The Journal of molecular diagnostics: JMD.

[29] Baddi L A (2005). Adjuvant therapy in stage II colon cancer: current approaches. The oncologist.

[30] Davis ME, Zuckerman JE, Choi CH, Seligson D, Tolcher A, Alabi CA, Yen Y, Heidel JD, Ribas A (2010). Evidence of RNAi in humans from systemically administered siRNA via targeted nanoparticles. Nature.

[31] Lanford RE, Hildebrandt-Eriksen ES, Petri A, Persson R, Lindow M, Munk ME, Kauppinen S, Orum H (2010). Therapeutic silencing of microRNA-122 in primates with chronic hepatitis C virus infection. Science.

[32] Wu CW, Ng SS, Dong YJ, Ng SC, Leung WW, Lee CW, Wong YN, Chan FK, Yu J, Sung JJ (2012). Detection of miR-92a and miR-21 in stool samples as potential screening biomarkers for colorectal cancer and polyps. Gut.

[33] Kanaan Z, Rai SN, Eichenberger MR, Roberts H, Keskey B, Pan J, Galandiuk S (2012). Plasma miR-21: a potential diagnostic marker of colorectal cancer. Annals of surgery.

[34] Schoof CR, Botelho EL, Izzotti A, Vasques Ldos R (2012). MicroRNAs in cancer treatment and prognosis. American journal of cancer research.

[35] Toiyama Y, Takahashi M, Hur K, Nagasaka T, Tanaka K, Inoue Y, Kusunoki M, Boland CR, Goel A (2013). Serum miR-21 as a diagnostic and prognostic biomarker in colorectal cancer. Journal of the National Cancer Institute.

[36] Seike M, Goto A, Okano T, Bowman ED, Schetter AJ, Horikawa I, Mathe EA, Jen J, Yang P, Sugimura H, Gemma A, Kudoh S, Croce CM (2009). MiR-21 is an EGFR-regulated anti-apoptotic factor in lung cancer in never-smokers. Proceedings of the National Academy of Sciences of the United States of America.

[37] Liu ZL, Wang H, Liu J, Wang ZX (2013). MicroRNA-21 (miR-21) expression promotes growth, metastasis, and chemo- or radioresistance in non-small cell lung cancer cells by targeting PTEN. Molecular and cellular biochemistry.

[38] Ren Y, Zhou X, Mei M, Yuan XB, Han L, Wang GX, Jia ZF, Xu P, Pu PY, Kang CS (2010). MicroRNA-21 inhibitor sensitizes human glioblastoma cells U251 (PTEN-mutant) and LN229 (PTEN-wild type) to taxol. BMC cancer.

[39] Mei M, Ren Y, Zhou X, Yuan XB, Han L, Wang GX, Jia Z, Pu PY, Kang CS, Yao Z (2010). Downregulation of miR-21 enhances chemotherapeutic effect of taxol in breast carcinoma cells. Technology in cancer research & treatment.

[40] Gaur AB, Holbeck SL, Colburn NH, Israel MA (2011). Downregulation of Pdcd4 by mir-21 facilitates glioblastoma proliferation *in vivo*. Neuro-oncology.

[41] Moriyama T, Ohuchida K, Mizumoto K, Yu J, Sato N, Nabae T, Takahata S, Toma H, Nagai E, Tanaka M (2009). MicroRNA-21 modulates biological functions of pancreatic cancer cells including their proliferation, invasion, and chemoresistance. Molecular cancer therapeutics.

[42] Liu M, Tang Q, Qiu M, Lang N, Li M, Zheng Y, Bi F (2011). miR-21 targets the tumor suppressor RhoB and regulates proliferation, invasion and apoptosis in colorectal cancer cells. FEBS letters.

[43] Bullock MD, Pickard KM, Nielsen BS, Sayan AE, Jenei V, Mellone M, Mitter R, Primrose JN, Thomas GJ, Packham GK, Mirenzami AH (2013). Pleiotropic actions of miR-21 highlight the critical role of deregulated stromal microRNAs during colorectal cancer progression. Cell death & disease.

[44] Kjersem JB, Ikdahl T, Lingjaerde OC, Guren T, Tveit KM, Kure EH (2014). Plasma microRNAs predicting clinical outcome in metastatic colorectal cancer patients receiving first-line oxaliplatin-based treatment. Molecular oncology.

[45] Koga Y, Yamazaki N, Yamamoto Y, Yamamoto S, Saito N, Kakugawa Y, Otake Y, Matsumoto M, Matsumura Y (2013). Fecal miR-106a is a useful marker for colorectal cancer patients with false-negative results in immunochemical fecal occult blood test. Cancer epidemiology, biomarkers & prevention : a publication of the American Association for Cancer Research, cosponsored by the American Society of Preventive Oncology.

[46] Feng B, Dong TT, Wang LL, Zhou HM, Zhao HC, Dong F, Zheng MH (2012). Colorectal cancer migration and invasion initiated by microRNA-106a. PloS one.

[47] Senanayake U, Das S, Vesely P, Alzoughbi W, Frohlich LF, Chowdhury P, Leuschner I, Hoefler G, Guertl B (2012). miR-192, miR-194, miR-215, miR-200c and miR-141 are downregulated and their common target ACVR2B is strongly expressed in renal childhood neoplasms. Carcinogenesis.

[48] Braun CJ, Zhang X, Savelyeva I, Wolff S, Moll UM, Schepeler T, Orntoft TF, Andersen CL, Dobbelstein M (2008). p53-Responsive micrornas 192 and 215 are capable of inducing cell cycle arrest. Cancer research.

[49] Karaayvaz M, Pal T, Song B, Zhang C, Georgakopoulos P, Mehmood S, Burke S, Shroyer K, Ju J (2011). Prognostic significance of miR-215 in colon cancer. Clinical colorectal cancer.

[50] Denby L, Ramdas V, Lu R, Conway BR, Grant JS, Dickinson B, Aurora AB, McClure JD, Kipgen D, Delles C, van Rooij E, Baker AH (2014). MicroRNA-214 antagonism protects against renal fibrosis. Journal of the American Society of Nephrology: JASN.

[51] Iizuka M, Ogawa T, Enomoto M, Motoyama H, Yoshizato K, Ikeda K, Kawada N (2012). Induction of microRNA-214-p in human and rodent liver fibrosis. Fibrogenesis & tissue repair.

[52] Klein CA (2008). Cancer. The metastasis cascade. Science.

[53] Blansfield JA, Caragacianu D, Alexander HR, Tangrea MA, Morita SY, Lorang D, Schafer P, Muller G, Stirling D, Royal RE, Libutti SK (2008). Combining agents that target the tumor microenvironment improves the efficacy of anticancer therapy. Clinical cancer research: an official journal of the American Association for Cancer Research.

[54] Boehm T, Folkman J, Browder T, O'Reilly MS (1997). Antiangiogenic therapy of experimental cancer does not induce acquired drug resistance. Nature.

[55] Villarroya-Beltri C, Gutierrez-Vazquez C, Sanchez-Cabo F, Perez-Hernandez D, Vazquez J, Martin-Cofreces N, Martinez-Herrera DJ, Pascual-Montano A, Mittelbrunn M, Sanchez-Madrid F (2013). Sumoylated hnRNPA2B1 controls the sorting of miRNAs into exosomes through binding to specific motifs. Nature communications.

[56] Roccaro AM, Sacco A, Maiso P, Azab AK, Tai YT, Reagan M, Azab F, Flores LM, Campigotto F, Weller E, Anderson KC, Scadden DT, Ghobrial IM (2013). BM mesenchymal stromal cell-derived exosomes facilitate multiple myeloma progression. The Journal of clinical investigation.

[57] Zhang Y, Liu D, Chen X, Li J, Li L, Bian Z, Sun F, Lu J, Yin Y, Cai X, Sun Q, Wang K, Ba Y (2010). Secreted monocytic miR-150 enhances targeted endothelial cell migration. Molecular cell.

[58] Chen X, Ba Y, Ma L, Cai X, Yin Y, Wang K, Guo J, Zhang Y, Chen J, Guo X, Li Q, Li X, Wang W (2008). Characterization of microRNAs in serum: a novel class of biomarkers for diagnosis of cancer and other diseases. Cell research.

[59] Mitchell PS, Parkin RK, Kroh EM, Fritz BR, Wyman SK, Pogosova-Agadjanyan EL, Peterson A, Noteboom J, O'Briant KC, Allen A, Lin DW, Urban N, Drescher CW (2008). Circulating microRNAs as stable blood-based markers for cancer detection. Proceedings of the National Academy of Sciences of the United States of America.

[60] Valadi H, Ekstrom K, Bossios A, Sjostrand M, Lee JJ, Lotvall JO (2007). Exosome-mediated transfer of mRNAs and microRNAs is a novel mechanism of genetic exchange between cells. Nature cell biology.

[61] Hida J, Yasutomi M, Maruyama T, Fujimoto K, Uchida T, Okuno K (1997). Lymph node metastases detected in the mesorectum distal to carcinoma of the rectum by the clearing method: justification of total mesorectal excision. Journal of the American College of Surgeons.

[62] Scott N, Jackson P, al-Jaberi T, Dixon MF, Quirke P, Finan PJ (1995). Total mesorectal excision and local recurrence: a study of tumour spread in the mesorectum distal to rectal cancer. The British journal of surgery.

[63] Association of Coloproctology of Great Britain and Ireland (2007). Guidelines for the management of colorectal cancer.

[64] Brazma A, Hingamp P, Quackenbush J, Sherlock G, Spellman P, Stoeckert C, Aach J, Ansorge W, Ball CA, Causton HC, Gaasterland T, Glenisson P, Holstege FC (2001). Minimum information about a microarray experiment (MIAME)-toward standards for microarray data. Nature genetics.

[65] McShane LM, Altman DG, Sauerbrei W, Taube SE, Gion M, Clark GM (2005). Reporting recommendations for tumor marker prognostic studies (REMARK). Journal of the National Cancer Institute.

